# Non‐SMC condensin I complex subunit H participates in anti‐programmed cell death‐1 resistance of clear cell renal cell carcinomas

**DOI:** 10.1111/cpr.13400

**Published:** 2023-01-15

**Authors:** Zhi Chen, Weiqiang Ruan, Chunhao Guo, Ke Chen, Le Li, Jihua Tian, Zhiquan Hu, Dan Peng, Xing Zeng

**Affiliations:** ^1^ Department of Geriatrics, Tongji Hospital, Tongji Medical College Huazhong University of Science and Technology Wuhan Hubei China; ^2^ Department of Urology, Tongji Hospital, Tongji Medical College Huazhong University of Science and Technology Wuhan Hubei China; ^3^ Department of Nuclear Medicine, Tongji Hospital, Tongji Medical College Huazhong University of Science and Technology Wuhan Hubei China

## Abstract

Non‐SMC condensin I complex subunit H (NCAPH) is reported to play an important role and be a poor prognostic factor in various cancers. However, the function and regulatory mechanism of NCAPH in clear cell renal cell carcinoma (ccRCC) remain unknown. The roles of NCAPH on ccRCC growth were detected in vitro and in vivo assays. The regulatory mechanism of NCAPH was explored by immunoprecipitation assay, ubiquitination assay, ChIP assay, RIP assay, luciferase reporter assay and RNA pull‐down assay. The role of NCAPH in immunoregulation also was explored by flow cytometry, T cell‐mediated tumour cell killing assay and immune‐competent mouse model. In this research, we displayed that NCAPH was upregulated in ccRCC and patients with elevated NCAPH expression had an undesirable prognosis. Functionally, NCAPH depletion restrained ccRCC growth in vitro and in vivo. The elevated NCAPH was attributed to FOXP3‐mediated transcription, FUS‐mediated transcription splicing and METTL3‐mediated m6A modification. Moreover, YTHDC1 promoted NCAPH mRNA nuclear export, and IGF2BP3 enhanced NCAPH mRNA stability in an m6A‐dependent manner. NCAPH increased PD‐L1 expression by inhibiting the degradation of β‐catenin in ccRCC cells, which further facilitated aerobic glycolysis and immune tolerance of ccRCC. Collectively, our findings display the vital function of NCAPH in ccRCC and uncover that NCAPH may be regarded as a potential therapeutic target to reverse the immune tolerance of ccRCC.

## Graphical abstract

FOXP3‐mediated transcription and FUS‐mediated splicing increases MCAPH expression. YTHDC1 induces non‐SMC condensin I complex subunit H (NCAPH) mRNA nuclear export and IGF2BP3 enhances NCAPH mRNA stability in an m6A‐dependent manner. The elevated NCAPH proteins then enhance the stability of β‐catenin and activate β‐catenin‐related signalling pathway, which further promotes aerobic glycolysis and immune escape of clear cell renal cell carcinoma.

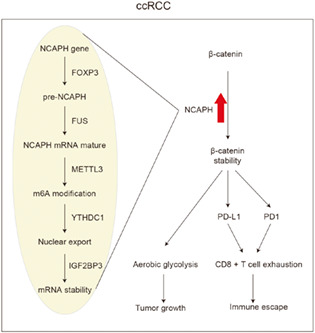

## 1 INTRODUCTION

Clear cell renal cell carcinoma (ccRCC) is the highest percentage histological subtype of kidney cancer.^1^ Despite great advances in the diagnosis and treatment of tumours, many patients are still diagnosed with metastases.^2^, ^3^ Surgical excision remains the optimal therapeutic strategy for ccRCC.^4^ In recent years, targeted therapy and immunotherapy have vastly extended the survival time of ccRCC patients.^5^, ^6^, ^7^ Nevertheless, the 5‐year survival rate for ccRCC remains very short.^8^ Thus, it is urgent to screen underlying biomarkers and therapeutic targets for the clinical diagnosis and treatment of ccRCC.

Non‐SMC condensin I complex subunit H (NCAPH) is one of the non‐SMC subunits in condensin I.^9^ NCAPH plays a crucial effect on mitotic chromosome architecture and segregation.^10^ NCAPH is increased in colon cancerous tissues and NCAPH depletion inhibits colon cancer growth, and migration, and induces cell apoptosis and cell cycle arrest.^11^ Wang et al. indicate that NCAPH is elevated in cervical cancer and positively correlated to prognosis.^12^ NCAPH inhibition restrains the proliferation, colony formation, migration, invasion and EMT process of cervical cancer cells by the PI3K/AKT/SGK pathway. Ogura et al. suggest that NCAPH is an undesirable prognostic factor in ER‐positive breast cancer patients and NCAPH depletion suppresses the growth of breast cancer cells.^13^ Xiong et al. display that NCAPH is elevated in non‐small cell lung cancer (NSCLC) and implicated in NSCLC progression by binding to β‐catenin protein and activating Wnt signalling.^14^ NCAPH is also reported to play an oncogene in bladder cancer,^15^ hepatocellular carcinoma,^16^ pancreatic cancer,^17^ lung cancer^18^, ^19^ and oral squamous cell carcinoma.^20^ However, the function of NCAPH in ccRCC was barely reported.

N6‐methyladenosine (m6A), an epigenetic modification of RNA, is related to RNA transcription splicing, transport, translation and degradation. As a reversible process, m6A modification is triggered by m6A methyltransferases and excised by m6A demethylases. The function of m6A modification is controlled by these proteins binding to m6A sites. There is mounting evidence indicating that m6A modification plays a key role in many human cancers, including ccRCC.^21^, ^22^, ^23^ Zhu et al. indicate that METTL3 promotes tumorigenesis via regulating HHLA2 mRNA expression in an m6A‐dependent manner in ccRCC.^24^ Besides, Shi et al. show that METTL3 promotes ABCD1 translation in an m6A‐dependent manner in ccRCC.^25^ However, the function and mechanism of m6A modification in ccRCC still need more exploration.

The presence of dysfunctional CD8+ T cells in the tumour microenvironment is a hallmark of low antitumor immune function.^26^ The prolonged presence of tumour antigens and/or inhibitory TME drives antitumor effector CD8+ T cells into a state of impaired function known as ‘T‐cell exhaustion’.^27^ Exhausted CD8+ T cells express high levels of inhibitory receptors, such as programmed cell death‐1 (PD1)^27^, ^28^ and the function of partially exhausted CD8+ T cells could be partially reversed by anti‐PD1 antibodies.^29^, ^30^ Wang et al. indicated that TOX promotes promote CD8+ T cell exhaustion by inhibiting PD1 degradation.^31^ Rong et al. showed that Matrix Gla protein promoted NF‐κB phosphorylation, thereby activating PD‐L1 expression to promote CD8+ T cell exhaustion.^32^ However, the molecular mechanisms that regulate CD8+ T‐cell exhaustion in TME remain largely unknown.

Here, NCAPH is found to be increased in ccRCC cell lines and tissues and accelerates ccRCC cell growth in vitro and in vivo. Elevated NCAPH level is induced by FOXP3‐mediated transcription and FUS‐mediated transcription splicing. The m6A modification of NCAPH increased its nuclear export and mRNA stability. Besides, NCAPH promotes aerobic glycolysis and PD‐L1 expression by inhibiting the degradation of β‐catenin. NCAPH inhibits the T cell response and induces resistance to anti‐PD‐1 therapy. Thus, NCAPH may be regarded as an oncogene and a latent immunomodulator in ccRCC.

## 2 MATERIALS AND METHODS

### 2.1 Human samples

A total of 87 paired tumour tissues and adjacent normal tissues were obtained from ccRCC patients who were diagnosed at Tongji Hospital, Tongji Medical College, Huazhong University of Science and Technology. The patient's clinical information is summarized in Table S1. This study was approved by the Medical Ethics Committee of Tongji Hospital, Tongji Medical College, Huazhong University of Science and Technology. Written informed consent was obtained.

### 2.2 Cell culture

Human Kidney‐2 (HK‐2) cells, human ccRCC cell lines (796‐P, 786‐O, ACHN and Caki‐1) and mouse Renca cells were bought from the ATCC and maintained in RPMI‐1640 medium supplemented with 10% FBS. All cells were verified by short tandem repeat and were checked for mycoplasma contamination.

### 2.3 Plasmids

The shRNAs targeting NCAPH, FOXP3, METTL3, YTHDC1, IGF2BP3 and overexpression plasmids of NCAPH, FOXP3, FUS, YTHDC1, IGF2BP3 and β‐catenin were all generated by Genepharma. Cell transfection was accomplished by a lentivirus‐based expression system. All the sequences for shRNAs were listed in Table S2.

### 2.4 RNA extraction and qRT‐PCR

Total RNA extraction and detection of target genes were executed as described previously.^33^ For the subcellular fractionation of RNA, cytoplasmic & nuclear RNA purification kit (Amyjet Scientific) was employed following the manual. The primers are listed in Table S2.

### 2.5 CCK‐8 assay

Cell viability was evaluated by Cell Counting Kit‐8 assay (Amyjet Scientific) as described previously.^33^

### 2.6 Colony formation assay

Transfected cells (1000 per well) were independently placed onto 6‐well plates. After 2 weeks of growth, cells were fixed with 4% paraformaldehyde and stained with crystal violet. The colonies (>40 μm) were counted.

### 2.7 5‐Ethynyl‐2′‐deoxyuridine

After transfection for 24 h, the cells were incubated with 50 μM 5‐Ethynyl‐2′‐deoxyuridine (EDU) (RIBOBIO) for 2 h and stained with DAPI. The number of EDU‐positive cells was counted.

### 2.8 Western blotting and immunoprecipitation

Total protein was extracted and the target protein was detected as described previously.^33^ For immunoprecipitation (IP), the lysate was precleared by protein A/G‐agarose, and IP was carried out by incubating the supernatants with antibodies against NCAPH or β‐catenin and protein A/G‐agarose overnight at 4°C. After centrifugation, the complexes were washed and resuspended. The targeting proteins were detected by Western blotting. Rabbit IgG isotype was used as a negative control. All antibody information was listed as follows: NCAPH (PA5‐80842, Invitrogen), β‐actin (ab8226, Abcam), FOXP3 (ab215206, Abcam), FUS (PA5‐52610, Invitrogen), METTL3 (ab195352, Abcam), YTHDC1 (ab259990, Abcam), IGF2BP1 (ab184305, Abcam), IGF2BP2 (ab248279, Abcam), IGF2BP3 (ab248279, Abcam), β‐catenin (ab6301, Abcam), PD‐L1 (ab213480, Abcam), Granzyme‐B (MA1‐80734, Invitrogen), Perforin (14‐9993‐82, Invitrogen).

### 2.9 Ubiquitination assay

For the ubiquitination assay, the indicated cells were transfected with various constructs together with Myc‐ubiquitin and Flag‐β‐catenin, treated with MG132, and lysed using RIPA lysis buffer. Ubiquitination was assessed by IP with an antibody against the Flag tag, followed by western blotting with an anti‐Myc antibody.

### 2.10 ChIP assay

ChIP assay was carried out as described previously.^33^ Lysates were immunoprecipitated with control IgG (ab172730, Abcam) and antibodies against FOXP3 (ab215206, Abcam). The immunoprecipitated DNA fragment was analysed by qRT‐PCR. The primers are listed in Table S2.

### 2.11 RIP assay

RIP assay was carried out as described previously.^33^ Antibodies against m6A (ab208577, Abcam), METTL3 (ab221795, Abcam), YTHDC1 (ab264375, Abcam), IGF2BP1 (ab229700, Abcam), IGF2BP2 (ab128175, Abcam), IGF2BP3 (ab250015, Abcam) and FUS (ab243880, Abcam) were used.

### 2.12 Luciferase reporter assay

The promoter of NCAPH (−2000 ~ 0) with mutated FOXP3 binding site was generated with QuickMutation™Plus Site‐Directed Mutagenesis Kit (Beyotime). Wild‐type and mutant promoter were synthesized and subcloned into a pGL3‐Basic luciferase reporter vector (Promega). All luciferase assays were analysed after 48 h of transfection using Dual‐Luciferase Kit (Promega). The luciferase activities were detected by a Dual‐Luciferase Reporter Assay System (Promega) following the manual.

### 2.13 Actinomycin D assay

Cells were treated with actinomycin D (10 μg/ml) for 0, 6 and 12 h. The expression of NCAPH mRNA in cells was determined by qRT‐PCR.

### 2.14 Flow cytometry

To measure apoptosis, ccRCC cells were double‐stained with annexin V‐FITC and PI (Beyotime) following the manual. Then stained cells were measured using a flow cytometer (Beckman Coulter).

To assess the level of membrane PD‐L1 in ccRCC cells and membrane PD‐1 in CD8‐T cells, cells were stained with Alexa Fluor® 488 Anti‐PD‐L1 antibody (ab209959, Abcam) or Alexa Fluor® 488 Anti‐PD1 antibody (ab220300, Abcam) and then measured using a flow cytometer.

### 2.15 T cell‐mediated tumour cell killing assay

Human CD8+ T cells were isolated from peripheral blood mononuclear cells by a CD8+ Cell Positive Selection Kit (ImunoSep, Precision BioMedicals Co., Ltd.) and activated with Dynabeads™ Human T‐Activator CD3/CD28 (Gibco) for 1 week following to the manual. ccRCC cells were seeded into 6‐well plates for 24 h and then activated CD8+ T cells were cocultured with adhered ccRCC cells for 48 h at a ratio of 3:1. After 48 h of incubation, cell debris was removed and CD8 T cells were collected. ccRCC cells were harvested for apoptosis detection by flow cytometry.

### 2.16 Enzyme‐linked immunosorbent assay

The level of IFN‐γ and TNF‐α produced by CD8+ T cells were measured by enzyme‐linked immunosorbent assay (ELISA) kits (eBioscience) following the manual.

### 2.17 Biotinylated RNA pull‐down assay

Pull‐down assay was carried out as previously described.^33^ The biotinylated NCAPH probe, biotinylated pre‐NCAPH probe and biotinylated NC probe were produced by GenePharma. The precipitated proteins were identified by Western blotting.

### 2.18 In vivo study

C57BL/6 and Balb/c nude mice were purchased from the Shanghai SLAC and housed under pathogen‐free conditions. The animal experiment was authorized by the Ethics Committee of Tongji Hospital, Tongji Medical College, Huazhong University of Science and Technology.

For the immunodeficient mouse model, NCAPH‐depleted human Caki‐1 cells (5 × 10^6^) and their control group were injected subcutaneously into Balb/c nude mice. Tumour sizes were measured and calculated as length × width^2^ × 0.5.

For the immune‐competent mouse model, NCAPH overexpressed Renca cells were injected subcutaneously into C57BL/6 mice. In vivo PD‐1 mAb treatments were conducted by intraperitoneal injection (100 μg per mouse in 100 μl D‐PBS buffer) every 4 days for up to four times. The tumour sizes and body weights were recorded. The tumour specimens were surgically removed, fixed, embedded in paraffin and sectioned. The sections were used for haematoxylin and eosin (H&E) and immunohistochemistry (IHC) staining.

### 2.19 Immunohistochemistry

The slides were deparaffinized, rehydrated, antigen retrieval and endogenous peroxidase blocking. After blocking with 10% goat serum, the tissues were incubated with antibodies against Ki67 or CD8 at 4°C overnight. After washing, the slides were incubated with a secondary antibody and HRP‐conjugated streptavidin in sequence. Then, DAB (Yeasen Biotechnology Co., Ltd.) was carried out to visualize targeting proteins. Haematoxylin was carried out to counterstain the slides.

### 2.20 Statistics

The data are presented as the mean ± SD. Data analysis was conducted using GraphPad Prism 7.0. Student's *t*‐tests and one‐way ANOVA with Tukey's post hoc test were used for difference analysis. Correlations analyses were evaluated by Pearson correlation coefficient. The survival data were analysed using the log‐rank test. All experiments were repeated at least three times. Differences with *p* < 0.05 were considered significant.

## 3 RESULTS

### 3.1 Elevated NCAPH level is related to poor prognosis in ccRCC patients

**FIGURE 1:**
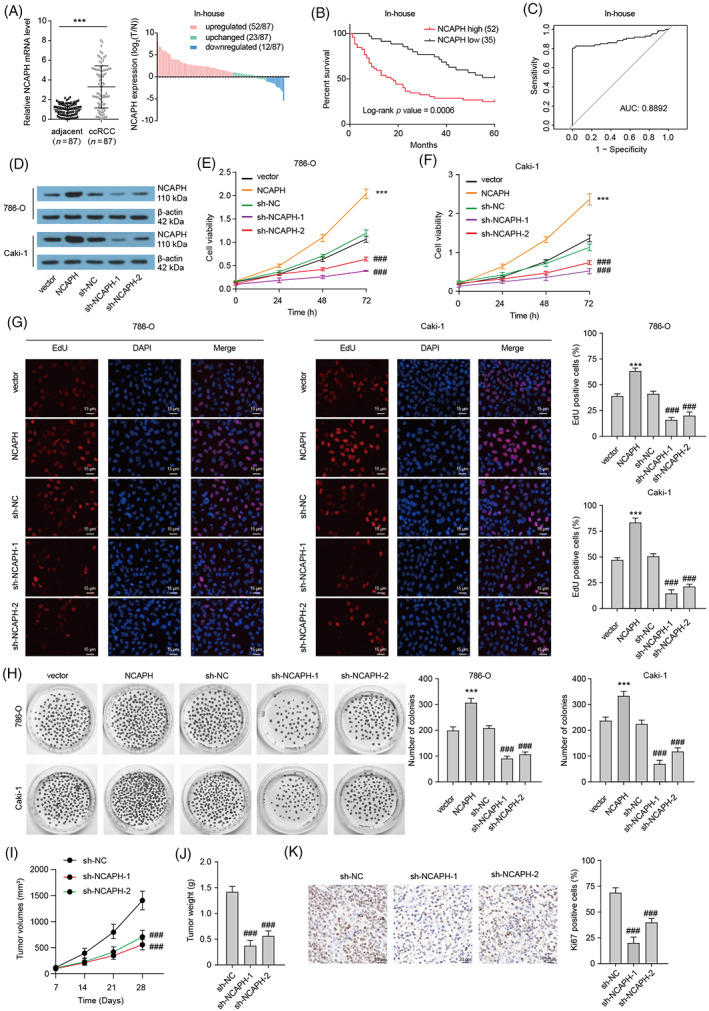
Elevated non‐SMC condensin I complex subunit H (NCAPH) promotes clear cell renal cell carcinoma (ccRCC) growth. (A) NCAPH expression in 87 paired ccRCC tissues and normal tissues was assessed by qRT‐PCR. Paired *t*‐test been used, ***p < 0.001. (B) Kaplan–Meier plot showing the relationship between NCAPH levels and patient overall survival. (C) ROS curve showing the potential diagnostic value in ccRCC. (D) NCAPH protein expression in 786‐O and Caki‐1 cells with NCAPH overexpression or depletion was assessed by Western bolting. (E and F) Cell viability of 786‐O and Caki‐1 cells with NCAPH overexpression or depletion was assessed by CCK‐8 assays. ***p < 0.001 versus vector; ^###^ *p* < 0.001 versus sh‐NC. (G) EDU staining shows the proliferative activity of 786‐O and Caki‐1 cells. ***p < 0.001 versus vector; ^###^ *p* < 0.001 versus sh‐NC. (H) Clone formation assay showing the clonal formation ability of 786‐O and Caki‐1 cells. ***p < 0.001 versus vector; ^###^ *p* < 0.001 versus sh‐NC. (I and J) Caki‐1 cells transfected with sh‐NC or sh‐NCAPH were injected into injected subcutaneously into Balb/c nude mice (five mice per group). The effect of NCAPH silencing on tumour volume curve and tumour weight was analysed. ^###^ *p* < 0.001 versus sh‐NC. (K) Ki67 staining of xenografts. ^###^ *p* < 0.001 versus sh‐NC. All experiments were repeated at least three times.

The level of NCAPH was found to be increased in a variety of tumours, including Kidney Renal Clear Cell Carcinoma (KIRC) by analysing The Cancer Genome Atlas (TCGA) data using TIMER (http://timer.cistrome.org/) (Figure S1A). CcRCC patients with elevated NCAPH expression had short overall survival time and disease‐free survival time (Figure S1B,C). NCAPH protein level was elevated in Clinical Proteomic Tumour Analysis Consortium (CPTAC, http://ualcan.path.uab.edu/analysis-prot.html) ccRCC samples when compared to the normal tissues (Figure S1D). We also confirmed the elevated NCAPH level in 87 paired ccRCC tissues from our hospital (Figure 1A). Increased NCAPH expression was closely related to large tumour size, higher histologic grades and advanced TNM stages (Table S1) and poor prognosis in ccRCC patients (Figure 1B). Receiver Operating Characteristic curve displayed that NCAPH may be used as a diagnostic indicator for ccRCC patients (Figure 1C). Finally, compared with HK‐2 cells, NCAPH mRNA and protein expression in ccRCC cell lines were significantly higher (Figure S1E,F). Collectively, our data indicate that NCAPH may play an oncogenic role in ccRCC.

### 3.2 NCAPH accelerates ccRCC cells growth in vitro and in vivo

To confirm the role of NCAPH in ccRCC, we constructed NCAPH overexpressed or depleted ccRCC cells (Figure 1D; Figure S1G,H). Results of CCK‐8 assay indicated that NCAPH overexpression significantly increased and NCAPH depletion obviously reduced the cell viability of 786‐O and Caki‐1 cells (Figure 1E,F). Furthermore, an EDU staining assay was used to evaluate the proliferation of ccRCC cells (Figure 1G). There were more EDU‐positive cells in NCAPH‐overexpressed ccRCC cells and lesser EDU‐positive cells in NCAPH‐depleted ccRCC cells. NCAPH‐overexpressed ccRCC cells showed greater ability to form clones, whereas NCAPH‐depleted ccRCC cells have weak clonal formation ability (Figure 1H). Then, a xenograft model was established to explore the function of NCAPH in vivo. NACPH depletion reduced the tumour volume and weight of xenograft (Figure 1I,J). Besides, lesser Ki67‐positive cells were observed in NCAPH‐depleted xenografts (Figure 1K). Taken together, our data strongly verify that NCAPH accelerates ccRCC cell growth in vitro and in vivo, and targeting NCAPH may be an underlying therapeutic strategy for ccRCC.

### 3.3 FOXP3 promotes the transcription and FUS facilitate the maturation of NCAPH

**FIGURE 2:**
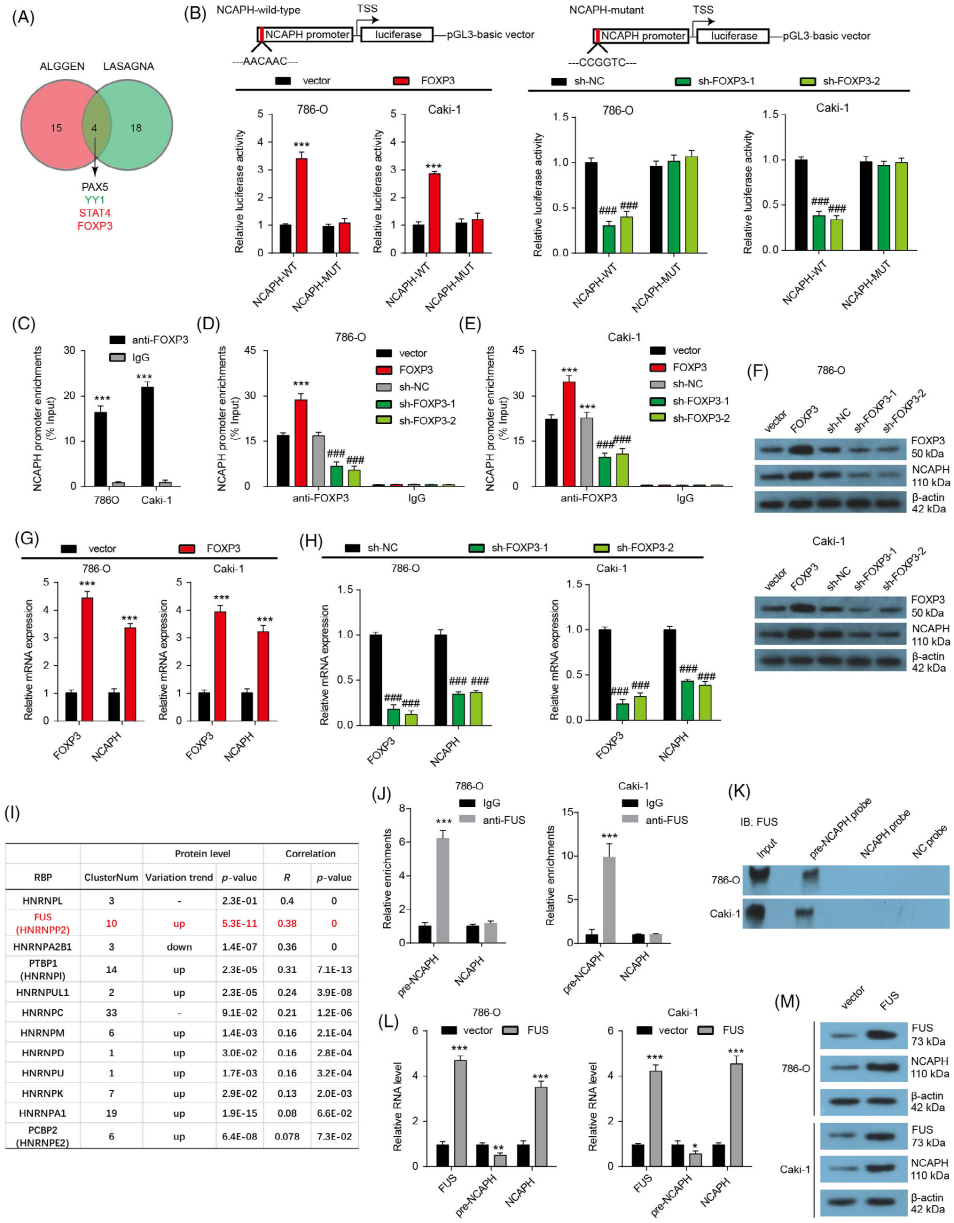
FOXP3 and FUS promote the transcription and maturation of non‐SMC condensin I complex subunit H (NCAPH) respectively. (A) Potential transcription factors for regulating NCAPH expression were predicted by ALGGEN and LASAGNA databases. (B) Luciferase reporter assays were carried out to verify the interaction between FOXP3 protein and NCAPH promoter. ***p < 0.001 versus vector; ^###^ *p* < 0.001 versus sh‐NC. (C) ChIP assays show the enrichment of FOXP3 protein in NCAPH promoter. ***p < 0.001 versus IgG. (D and E) FOXP3 overexpression or depletion changed the enrichment of FOXP3 protein in NCAPH promoter. ***p < 0.001 versus vector; ^###^ *p* < 0.001 versus sh‐NC. (F) The effect of FOXP3 on NCAPH protein expression was detected by Western bolting in 786‐O and Caki‐1 cells. (G and H) The effect of FOXP3 on NCAPH mRNA expression was detected by Western bolting in 786‐O and Caki‐1 cells. ***p < 0.001 versus vector; ^###^ *p* < 0.001 versus sh‐NC. (I) The table listed the hnRNPs which were predicted to bind to NCAPH by the ENCORI database. (J) RIP assay was used to verify the interaction between FUS protein and pre‐NCAPH. ***p < 0.001. (K) Pull‐down assay was further carried out by pre‐NCAPH probes, NCAPH probes, and NC probes to verify the interaction between FUS protein and pre‐NCAPH. (L) The effect of FUS on pre‐NCAPH and NCAPH mRNA expression. ***p < 0.001 versus vector. (M) The effect of FUS on NCAPH protein expression. Each experiment was repeated at least three times.

To explore the underlying mechanism for the overexpression of NCAPH in ccRCC, we predicted the underlying transcription factors by the ALGGEN‐PROMO database (http://alggen.lsi.upc.es/cgibin/promo_v3/promo/promoinit.cgi?dirDB=TF_8.3) and LASAGNA database (Length‐Aware Site Alignment Guided by Nucleotide Association, https://biogrid-lasagna.engr.uconn.edu/lasagna_search/) and four transcription factors were overlapped (Figure 2A). Among these four transcription factors, STAT4 and FOXP3 were upregulated, and YY1 was downregulated in TCGA KIRC samples, whereas there was no significant change of PAX5 in TCGA KIRC samples when compared with normal tissues (Figure S2A). Both STAT4 and FOXP3 levels were positively correlated to the expression of NCAPH in KIRC samples (Figure S2B,C). Patients with high FOXP3 expression showed poor prognoses (Figure S2D). However, there was no noteworthy association between STAT4 level and prognosis in KIRC (Figure S2E). Therefore, we chose FOXP3 as a further research object. Luciferase reporter assays showed that the overexpression of FOXP3 increased the luciferase activity of the wild‐type but not the mutant construct (Figure 2B). FOXP3 depletion plays the opposite role. ChIP assays indicated that FOXP3 could occupy the promoter of NCAPH (Figure 2C). FOXP3 overexpression increased the enrichment of FOXP3 in NCAPH promoter, and upregulated the mRNA and protein expression of NCAPH (Figure 2D–H). These results affirm that FOXP3 promotes the transcription of NCAPH.

Heterogeneous nuclear ribonucleoproteins (hnRNPs) are reported to control the alternative splicing of RNAs and assist mRNAs transport, translation and stabilization. Here, we filtrated the hnRNPs which were predicted to binding to NCAPH by using ENCORI (The Encyclopedia of RNA Interactomes, https://starbase.sysu.edu.cn/) database and 12 hnRNPs were screened out (Figure 2I). The change of 12 hnRNPs expression and the correlation between hnRNPs and NCAPH in KIRC were analysed. Nine hnRNPs were found to be up‐regulated and only one hnRNPs was found to be down‐regulated in ccRCC samples. Next, correlation analysis indicated that seven up‐regulated hnRNPs were found to be positively correlated to NCAPH expression in ccRCC samples. Among the seven filtrated hnRNPs, FUS was selected for its highest *R*‐value. FUS was predicted to binding to the 3′UTR and introns of NCAPH. RIP and Pulldown assays affirmed that FUS could bind to precursor NCAPH (pre‐NCAPH), but not NCAPH mRNA in both ccRCC cell lines (Figure 2J,K). Moreover, FUS overexpression significantly reduced the pre‐NCAPH levels, enhanced NCAPH mRNA levels and increased NCAPH protein expression in ccRCC cells (Figure 2L,M). In short, FUS promotes the maturation of NCAPH mRNA and enhances the expression of NCAPH.

### 3.4 YTHDC1 induces NCAPH mRNA nuclear export and IGF2BP3 enhances NCAPH mRNA stability in an m6A‐dependent manner

**FIGURE 3:**
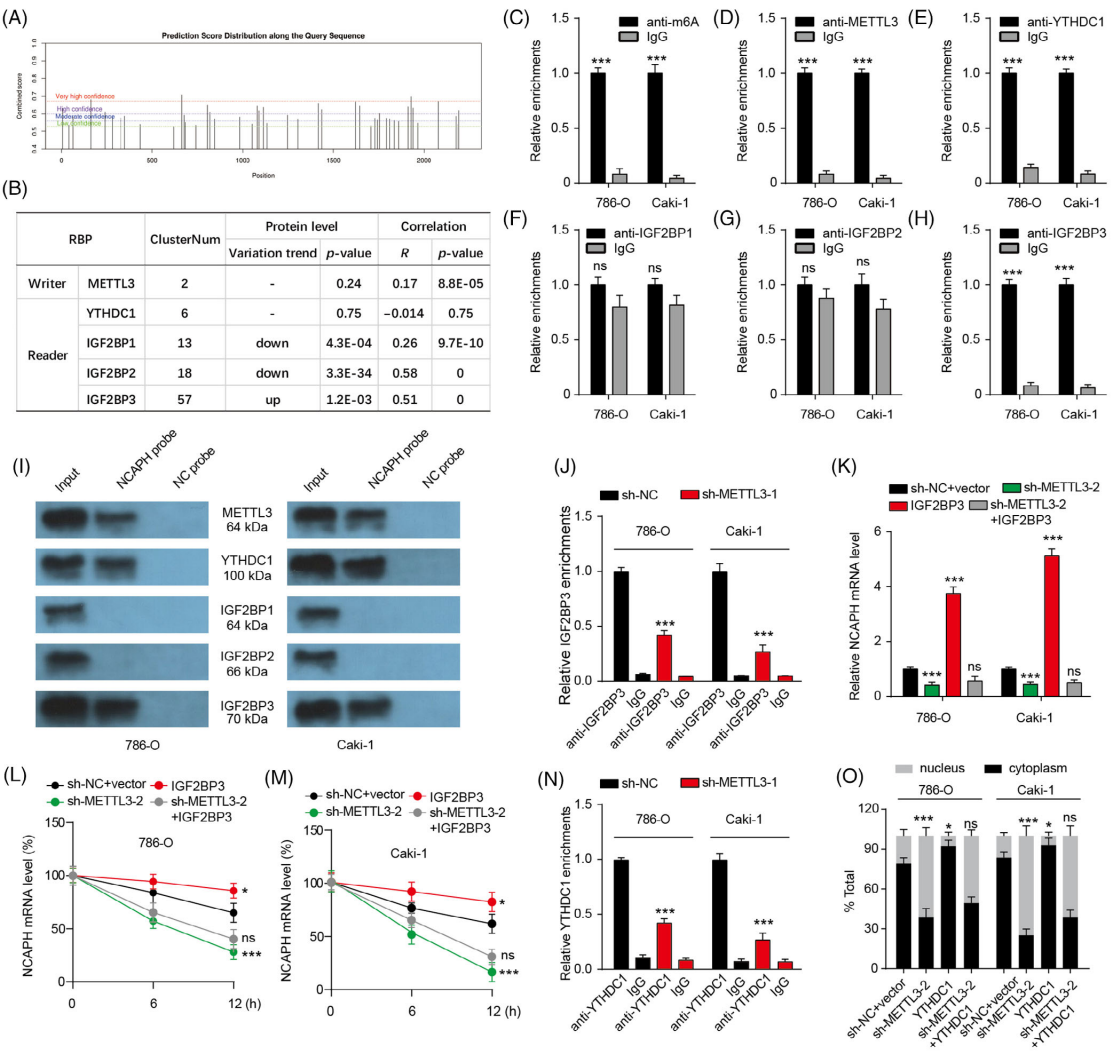
m6A modification of non‐SMC condensin I complex subunit H (NCAPH) mRNA affects its nuclear export and stability. (A) The latent modification sites for m6A in NCAPH mRNA were predicted by SRAMP (sequence‐based RNA adenosine methylation site predictor) database. (B) The table listed the potential binding RBPs which may regulate m6A modification of NCAPH mRNA. (C–H) RIP assays were used to verify the potential interaction between NCAPH mRNA and m6A Writers or Readers. ns *p* > 0.05; ***p < 0.001 versus IgG. (I) Pull‐down assays were used to verify the potential interaction between NCAPH mRNA and m6A Writers or Readers. (J) RIP assays show the effect of METTL3 depletion on the binding between NCAPH mRNA and IGF2BP3. ***p < 0.001 versus sh‐NC. (K) NCAPH expression in clear cell renal cell carcinoma (ccRCC) cells with METTL3 depletion or IGF2BP3 overexpression. ***p < 0.001 versus sh‐NC + vector; ns *p* > 0.05 versus sh‐METTL3‐2. (L and M) Time‐course qRT‐PCR analyses of the relative abundance of NCAPH in ccRCC cells with METTL3 depletion or IGF2BP3 overexpression treated with actinomycin D (10 μg/ml). *p < 0.05; ***p < 0.001 versus sh‐NC + vector; ns *p* > 0.05 versus sh‐METTL3‐2. (N) RIP assays show the effect of METTL3 depletion on the binding between NCAPH mRNA and YTHDC1. ***p < 0.001 versus sh‐NC. (O) The distribution of NCAPH mRNA in nucleus and cytoplasm in ccRCC cells with METTL3 depletion or YTHDC1 overexpression. *p < 0.05; ***p < 0.001 versus sh‐NC + vector; ns *p* > 0.05 versus sh‐METTL3‐2. Each experiment was repeated at least three times.

By analysing the sequence of NCAPH mRNA, multiple potential sites for m6A were found (Figure 3A). Moreover, m6A writer METTL3 and m6A reader IGF2BP1, IGF2BP2, IGF2BP3 and YTHDC1 were predicted to binding to NCAPH by ENCORI (Figure 3B). The protein level of IGF2BP1 and IGF2BP2 was downregulated, whereas IGF2BP3 protein level was upregulated in CPTAC ccRCC samples. There was no change in METTL3 and YTHDC1 protein expression. Correlation analysis showed that METTL3, IGF2BP1, IGF2BP2 and IGF2BP3 expression were correlated to NCAPH expression. Then, RIP assays were used to verify the m6A modification of NCAPH mRNA and the binding between NCAPH mRNA and m6A‐related proteins. As shown in Figure 3C, NCAPH mRNA could be immune‐precipitated by anti‐m6A antibodies, indicating that there was m6A modification in NCAPH mRNA. Moreover, NCAPH mRNA also could be immune‐precipitated by anti‐METTL3, anti‐YTHDC1 and anti‐IGF2BP3 antibodies, whereas NCAPH mRNA could not be immune‐precipitated by anti‐IGF2BP1 and anti‐IGF2BP2 antibodies (Figure 3D–H). Pull‐down assays by NCAPH probes further affirmed the interaction between NCAPH mRNA and METTL3, YTHDC1 and IGF2BP3 in both ccRCC cell lines (Figure 3I). Knockdown of METTL3 significantly reduced the m6A modification in NCAPH mRNA (Figure S3A). Moreover, METTL3 depletion markedly promoted NCAPH mRNA degradation and decreased NCAPH mRNA levels (Figure S3B,C). Similar to METTL3 depletion, IGF2BP3 depletion also was found to reduce the stability of NCAPH mRNA and inhibit NCAPH mRNA expression (Figure S3D,E). However, YTHDC1 depletion did not affect the stability and expression of NCAPH mRNA (Figure S3F,G). Given the nuclear export function of YTHDC1, we detected the distribution of NCAPH mRNA after YTHDC1 depletion and found a clear reduction of cytoplasmic NCAPH mRNA levels in ccRCC cells upon YTHDC1 depletion (Figure S3H), indicating that YTHDC1 was implicated in the nuclear export of NCAPH mRNA.

We then wonder whether IGF2BP3‐induced stability of NCAPH mRNA and YTHDC1‐induced nuclear export of NCAPH mRNA was related to METTL3‐mediated m6A modification of NCAPH mRNA. METTL3 depletion reduced the interaction between IGF2BP3 and NCAPH mRNA (Figure 3J). IGF2BP3 overexpression markedly increased NCAPH mRNA expression, whereas IGF2BP3 overexpression could not rescue the NCAPH expression upon METTL3 knockdown (Figure 3K). IGF2BP3 overexpression increased NCAPH mRNA stability and could not reverse the decreased NCAPH stability mediated by METTL3 knockdown (Figure 3L,M). These results indicate that NCAPH mRNA stability and expression were upregulated through the METTL3‐IGF2BP3 axis in an m6A‐dependent manner. Moreover, METTL3 depletion reduced the interaction between YTHDC1 and NCAPH mRNA and YTHDC1 overexpression could not rescue the reduction of cytoplasmic NCAPH mRNA upon METTL3 knockdown (Figure 3N,O), suggesting that YTHDC1‐induced nuclear export of NCAPH mRNA was dependent on METTL3‐mediated m6A modification.

### 3.5 NCAPH promotes PD‐L1 expression by inhibiting the degradation of β‐catenin

**FIGURE 4:**
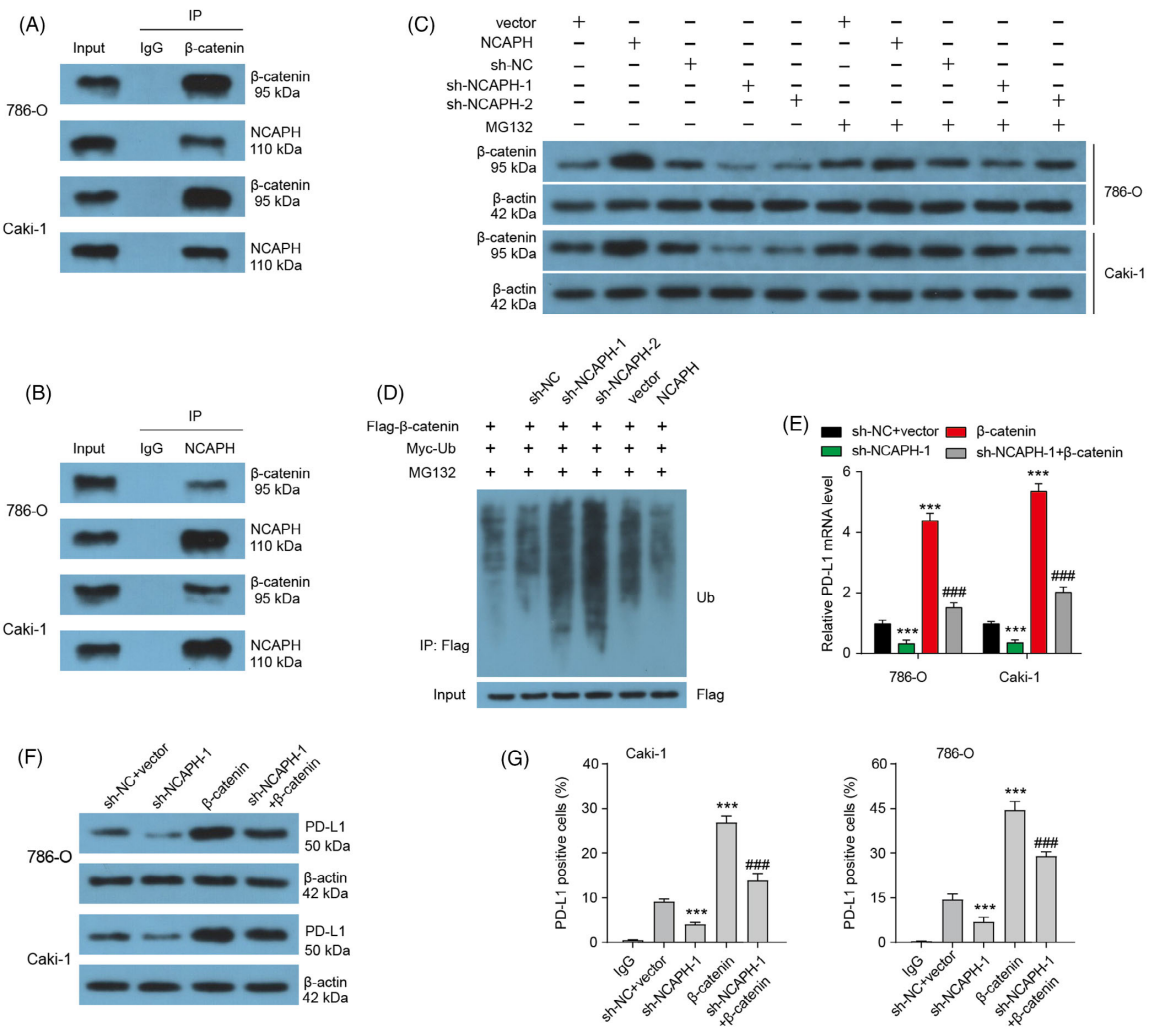
Non‐SMC condensin I complex subunit H (NCAPH) affects PD‐L1 expression by regulating the stability of β‐catenin protein. (A and B) The co‐IP assay was performed to detect the endogenous protein interaction between NCAPH and β‐catenin in 786‐O and Caki‐1 cells. (C) The treatment of MG132 blocked the change of β‐catenin level mediated by NCAPH overexpression or depletion. (D) Ubiquitination assay of β‐catenin in Caki‐1 cells co‐transfected with Myc‐Ub and Flag‐β‐catenin and treated with MG132. (E) PD‐L1 mRNA expression in clear cell renal cell carcinoma (ccRCC) cells was measured after NCAPH depletion or β‐catenin overexpression. ***p < 0.001 versus sh‐NC + vector; ^###^ *p* < 0.001 versus sh‐NCAPH‐1. (F) The PD‐L1 protein level in ccRCC cells was measured after NCAPH depletion or β‐catenin overexpression. (G) Membrane PD‐L1 level in ccRCC cells was measured after NCAPH depletion or β‐catenin overexpression by flow cytometry. Each experiment was repeated at least three times.

It is reported that NCAPH can inhibit ubiquitin‐mediated β‐catenin protein degradation in NSCLC.^14^ We then wondered whether NCAPH could regulate β‐catenin protein stability in ccRCC. To test the idea, co‐IP assays were carried out, and found that endogenous NCAPH proteins interact with β‐catenin proteins physically (Figure 4A,B). Moreover, NCAPH‐induced changed β‐catenin proteins were abolished by proteasome inhibitor MG132 treatment (Figure 4C). NCAPH overexpression decreased and NCAPH depletion increased the ubiquitin modification of β‐catenin (Figure 4D). These results indicate that NCAPH inhibits ubiquitin‐mediated β‐catenin protein degradation in ccRCC cells.

PD‐L1 is reported to be a target gene of β‐catenin in glioblastoma.^34^ Here, we found that β‐catenin overexpression significantly increased the mRNA and protein level of PD‐L1, as well as the membrane PD‐L1 in ccRCC cells (Figure 4E–G). NCAPH depletion has the opposite effect. β‐catenin overexpression could rescue the decreased PD‐L1 mRNA, PD‐L1 protein and membrane PD‐L1 level mediated by NCAPH knockdown. In other word, NCAPH regulates PD‐L1 expression by inhibiting the degradation of β‐catenin.

### 3.6 NCAPH promotes aerobic glycolysis by enhancing β‐catenin

**FIGURE 5:**
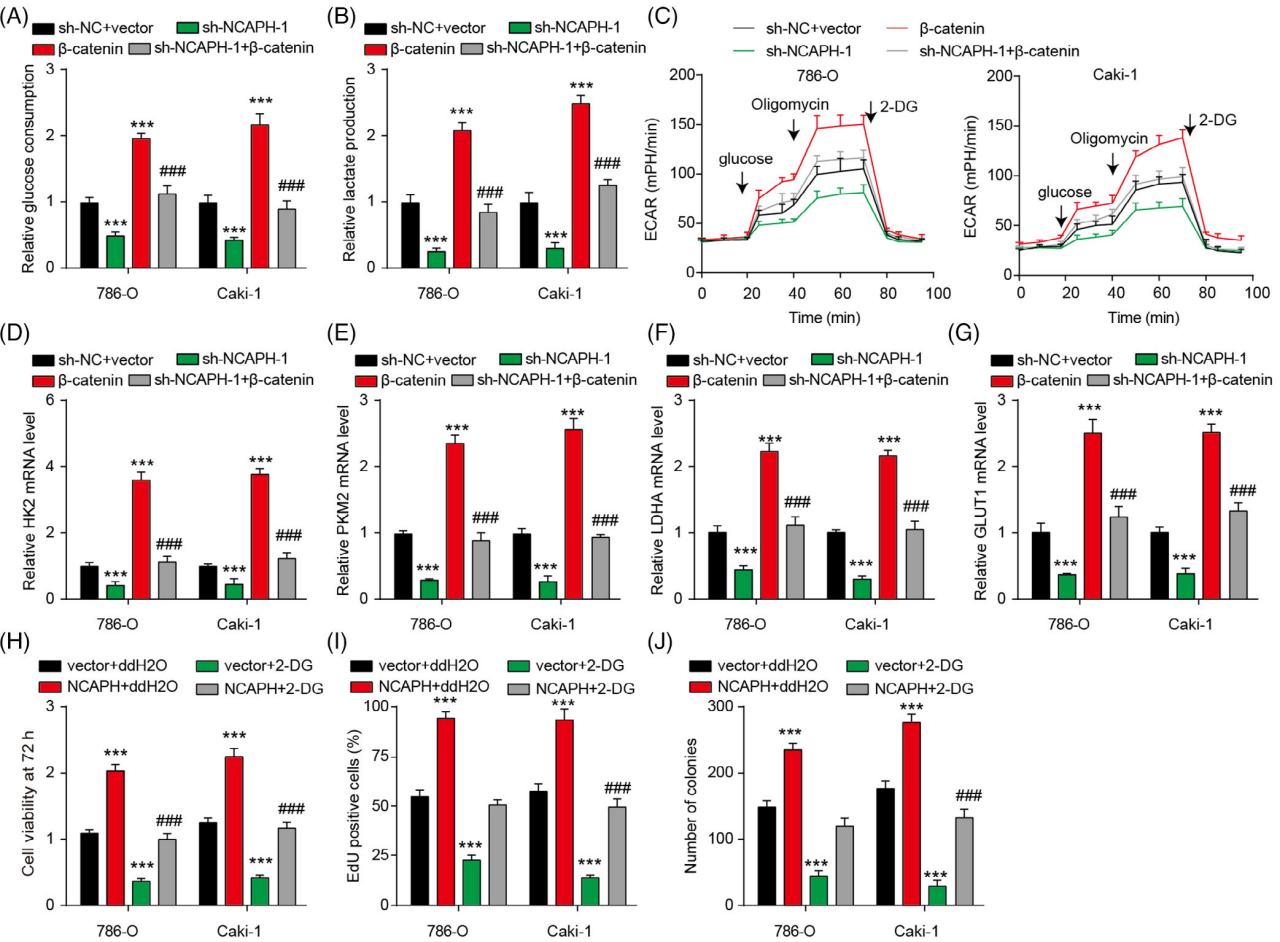
Non‐SMC condensin I complex subunit H (NCAPH) is related to aerobic glycolysis in clear cell renal cell carcinoma (ccRCC). (A and B) Glucose consumption and lactate production of ccRCC cells was measured. (C) The effect of NCAPH‐β‐catenin axis on the extracellular acidification rate (ECAR) in 786‐O and Caki‐1 cells. (D–G) The effect of NCAPH‐β‐catenin axis on the mRNA level of glycolysis‐related genes (HK2, PKM2, LDHA and GLUT1) in ccRCC cells. ***p < 0.001 vssh‐NC + vector; ^###^ *p* < 0.001 versus sh‐NCAPH‐1. (H) Cell viability of ccRCC cells was measured by CCK‐8 assay after NCAPH overexpression or 2‐DG treatment. (I) EDU‐positive cells were counted in ccRCC cells after NCAPH overexpression or 2‐DG treatment. (J) The number of clones was counted in ccRCC cells with NCAPH overexpression or 2‐DG treatment. ***p < 0.001 versus vector + ddH2O; ^###^ *p* < 0.001 versus NCAPH + ddH2O. Each experiment was repeated at least three times.

As β‐catenin is closely related to aerobic glycolysis in various tumours, we next explored whether NCAPH participated in aerobic glycolysis of ccRCC cells. NCAPH depletion obviously inhibited glucose consumption, lactate production, and ECAR in ccRCC cells (Figure 5A–C). NCAPH depletion also reduced the glycolysis‐related genes (HK2, PKM2, LDHA, GLUT1) expression (Figure 5D–G). β‐catenin overexpression rescued the decrease of glucose consumption, lactate production, ECAR, and glycolysis‐related gene expression mediated by NCAPH depletion. Moreover, the treatment of 2‐DG, an inhibitor for glycolysis, could decrease the cell viability, proliferative activity and colony‐forming ability of both ccRCC cell lines (Figure 5H–J). The treatment of 2‐DG reversed the cell viability, proliferative activity and colony‐forming ability of ccRCC cells induced by NCAPH overexpression. Thus, NCAPH promotes the growth of ccRCC cells by increasing β‐catenin expression and activating aerobic glycolysis.

### 3.7 NCAPH inhibits the T cell response against ccRCC

**FIGURE 6:**
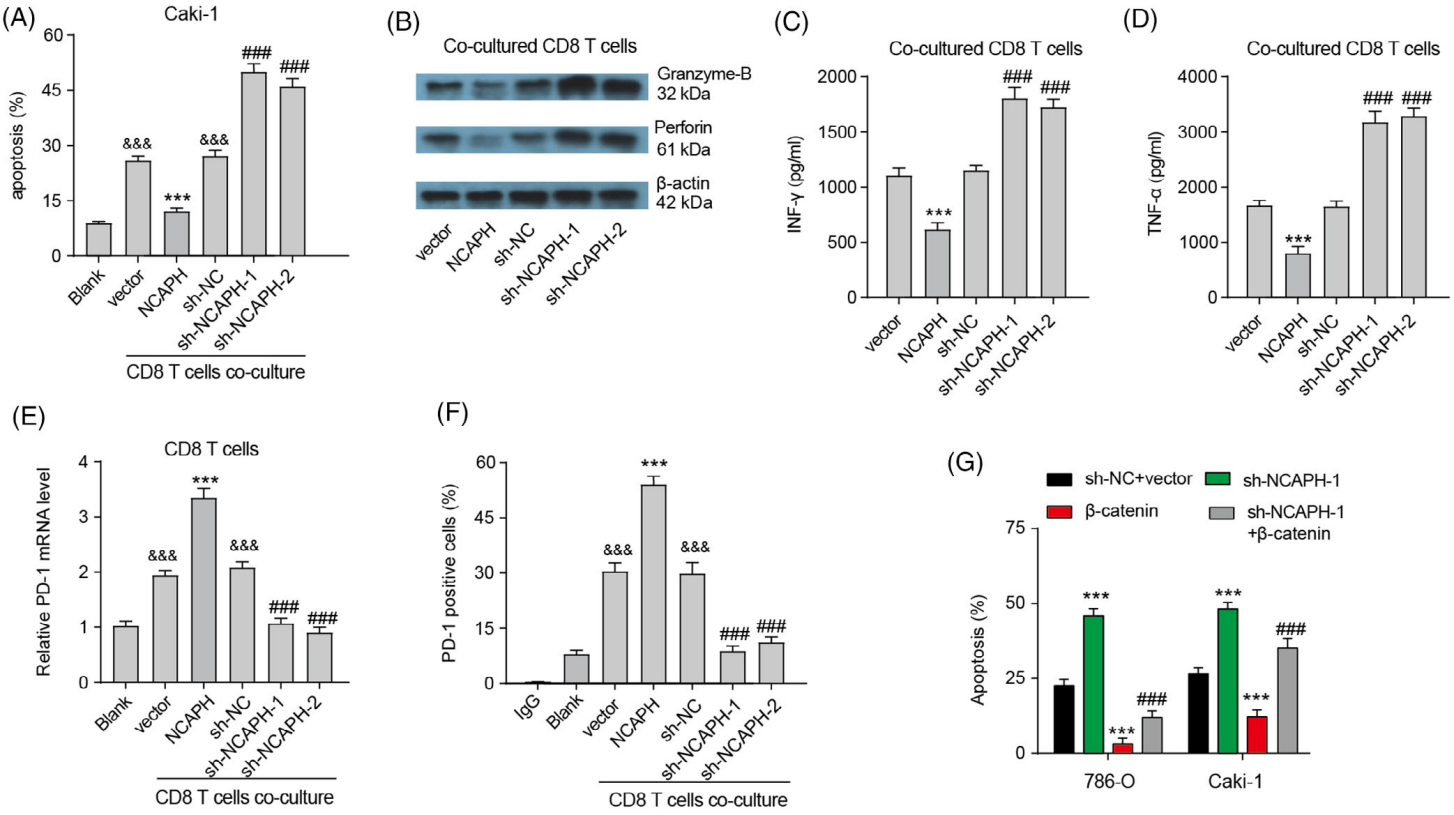
Non‐SMC condensin I complex subunit H (NCAPH) regulates the cytolysis of CD8 T cells through β‐catenin. (A) FACS analysis of CD8 T cell‐mediated elimination of Caki‐1 cells. ^&&&^ *p* < 0.001 versus Blank; ***p < 0.001 versus vector; ^###^ *p* < 0.001 versus sh‐NC. (B) Expression of perforin and Granzyme‐B in CD8 T cells after co‐cultured Caki‐1 cells with NCAPH overexpression or depletion was detected by Western bolting. (C–D) The levels of IFN‐γ and TNF‐α secreted by CD8 T cells were measured by ELISA assay. ***p < 0.001 versus vector; ^###^ *p* < 0.001 versus sh‐NC. (E) PD‐1 mRNA expression in CD8 T cells was measured. ^&&&^ *p* < 0.001 versus Blank; ***p < 0.001 versus vector; ^###^ *p* < 0.001 versus sh‐NC. (F) Membrane PD‐1 expression in CD8 T cells was measured by flow cytometry. ^&&&^ *p* < 0.001 versus Blank; ***p < 0.001 versus vector; ^###^ *p* < 0.001 versus sh‐NC. (G) CD8 T cell‐mediated elimination of clear cell renal cell carcinoma cells was measured by flow cytometry after NCAPH depletion or β‐catenin overexpression. ***p < 0.001 versus sh‐NC + vector; ^###^ *p* < 0.001 versus sh‐NCAPH‐1. Each experiment was repeated at least three times.

By using TISIDB, we found that NCAPH expression was significantly correlated to multiple TILs (tumour‐infiltrating lymphocytes), immunoinhibitors, immunostimulators, MHCs, chemokines and receptors (Figure S4A–F), indicating the potential immune regulatory function of NCAPH. In addition, NCAPH level was positively related to the infiltrated CD8 T cells, Tregs and macrophages in TIMER (Figure S4G). Here, we aimed to explore the influence of NCAPH on CD8 T cells. T cell‐mediated killing assays indicated that NCAPH depletion sensitized Caki‐1 cells to CD8 T cells‐mediated cytolysis, whereas overexpression decreased the cytolysis of CD8 T cells (Figure 6A). Moreover, the protein level of perforin, granzyme B and secreted IFN‐γ and TNF‐α from cocultured CD8 T cells was decreased by NCAPH overexpression and increased by NCAPH depletion (Figure 6B–D). As increased PD1 is closely related to CD8 T cell exhaustion, we then detected PD1 levels in cocultured CD8 T cells. NCAPH overexpression increased and NCAPH depletion inhibited the PD1 level in cocultured CD8 T cells (Figure 6E). Membrane PD1 expression also changes accordingly (Figure 6F). Besides, β‐catenin overexpression rescued the cytolysis of CD8 T cells promoted by NCAPH depletion (Figure 6G), indicating that NCAPH affects the cytolysis of CD8 T cells by regulating β‐catenin.

### 3.8 NCAPH induces resistance to anti‐PD‐1 therapy

**FIGURE 7:**
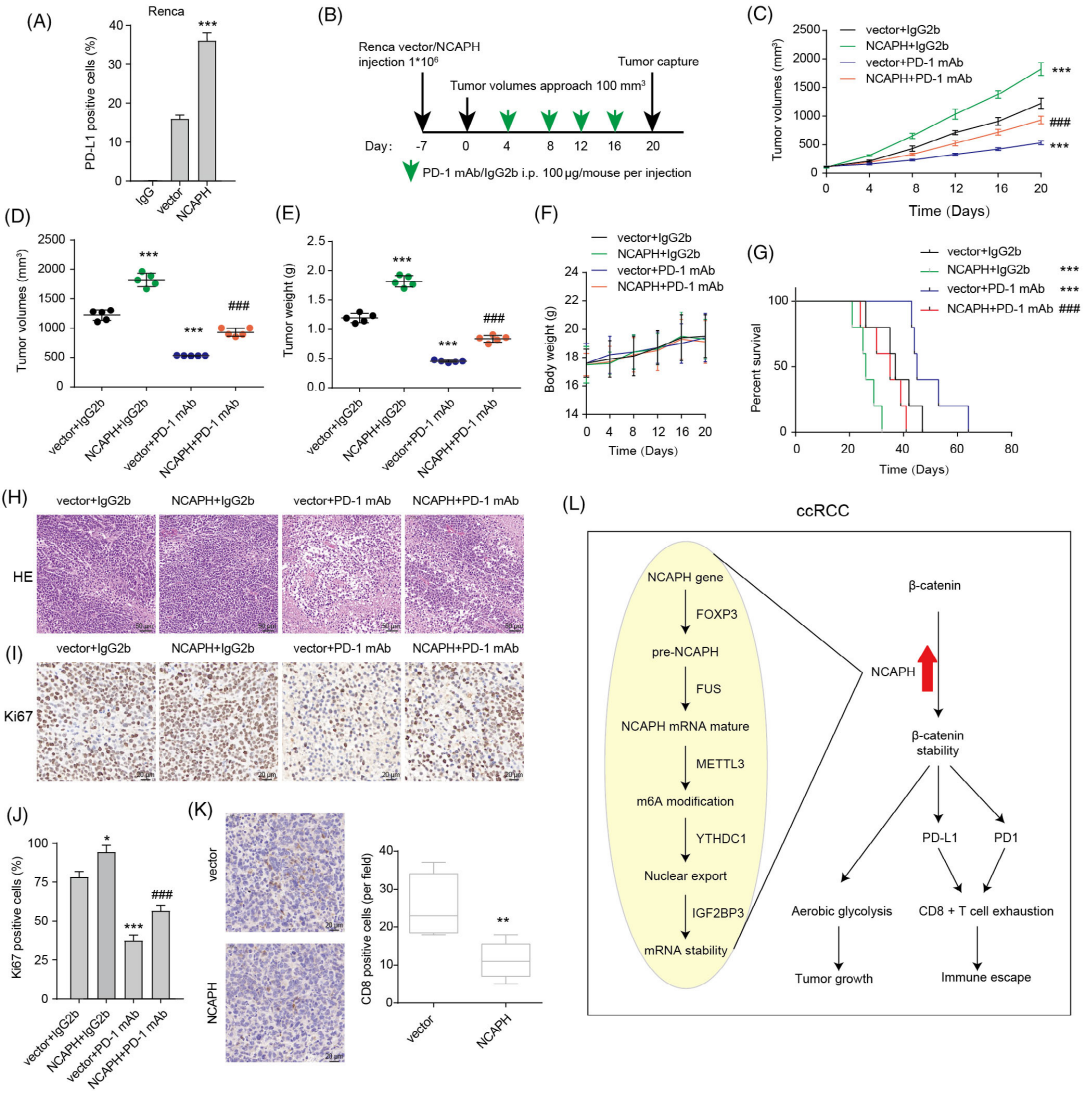
Non‐SMC condensin I complex subunit H (NCAPH) is related to the resistance to anti‐PD‐1 therapy in clear cell renal cell carcinoma (ccRCC). (A) Membrane PD‐1 expression in Renca cells with mouse NCAPH overexpression was measured by flow cytometry. (B) Schematic diagram showing the grouping and treatment plan of the in vivo study: C57BL/6 mice were inoculated with 10^6^ control or mouse NCAPH overexpressed Renca cells and received PD‐1 mAb treatment or IgG2b control at the indicated time points (five mice per group). (C) Tumour growth curves of the xenograft. (D and E) The volumes and weight of captured xenograft. (F) Body weight of mouse in the process of the treatment. (G) Survival curves of mouse with indicated treatment. (H and I) HE and Ki67 staining of xenograft. (J) The Ki67‐positive cells in the slice were counted. *p < 0.05; ***p < 0.001 versus vector + IgG2b; ^###^ *p* < 0.001 versus vector + PD‐1 mAb. (K) The infiltrated CD8 T cells were counted through CD8 IHC. **p < 0.01. (L) Working model for NCAPH/β‐catenin axis in ccRCC. Three independent experiments were performed.

NCAPH overexpression significantly increased membrane PD‐L1 expression (Figure 7A). To affirm the immunosuppressive function of NCAPH in vivo, we inoculated NCAPH‐overexpressing Renca cells into immunocompetent C57BL/6 mice with or without administration of PD1 mAb (Figure 7B). NCAPH overexpression promoted tumour growth, decreased the survival time, did not affect body weight, and inhibited CD8+ cell infiltration (Figure 7C–K). PD‐1 mAb inhibited tumour growth and increased the survival time. The xenograft mice with high NCAPH expression were resistant to anti‐PD‐1 therapy. In other word, NCAPH is involved in anti‐PD‐1 therapy resistance of ccRCC partly by regulating the dysfunction of CD8+ T cells.

## 4 DISCUSSION

Multiple types of biomarkers for ccRCC have been proposed and experimentally evaluated in the past decades.^35^, ^36^, ^37^ In this study, we identified that NCAPH was highly expressed in ccRCC and significantly correlated with clinical stages and prognosis. Overexpression of NCAPH could accelerate ccRCC cell growth and the dysfunction of CD8+ T cells in vitro and in vivo. Mechanistically, NCAPH enhanced PD‐L1 expression by inhibiting the degradation of β‐catenin.

Transcriptional control is an important gene expression. Wang et al. find that HPV E7 increases NCAPH gene expression via E2F1‐mediated transcription activation in cervical carcinoma.^12^ Moreover, Octamer transcription factor 1 (OCT1) is identified to be an activating transcription factor for NCAPH in breast cancer^13^ and MYB Proto‐Oncogene Like 2 (MYBL2) also is identified to be an activating transcription factor for NCAPH in NSCLC.^19^ Here, we found that transcription factor FOXP3, which was increased in ccRCC, could bind to NCAPH promoter and promote NCAPH transcription.

FUS, an RNA‐binding protein, was recently reported to be a novel regulator of the biogenesis of RNA.^38^ Satoshi et al. indicate that FUS regulates precursor mRNA splicing by interacting with beta‐catenin.^39^ Yang et al. suggest that FUS directly binds to precursor RHOBTB3 mRNA to promote the biogenesis of circRHOBTB3.^40^ In this study, we identified that NCAPH was a target for the alternative splicing factor FUS, which promoted the maturation of NCAPH mRNA by binding to precursor NCAPH. Therefore, our research revealed a new target of FUS.

The gene expression regulation network is very complex and is involved in multiple signalling pathways. In this study, we found that the abnormal expression of NCAPH in ccRCC is related to transcriptional control, alternative splicing and m6A modification. First, the transcription factor FOXP3 increased the transcription of NCAPH gene. Then, the mature NCAPH was generated by precursor NCAPH with the help of FUS. Besides, the m6A modification induced by METTL3 further enhanced NCAPH mRNA stability in an IGF2BP3‐dependent manner. Our study initially revealed the molecular mechanism of the abnormal expression of NCAPH in ccRCC, further expanded the regulatory mechanism of NCAPH, and laid a foundation for the treatment of ccRCC targeting NCAPH.

ccRCC is highly immune infiltrated and immune checkpoint blockade therapy and combination regimens have significantly improved the prognosis of ccRCC patients.^41^, ^42^ The infiltration of exhaustion‐phenotype CD8+ T cells is negatively correlated to ccRCC patient prognosis.^43^ Li et al. indicate that NCAPH is positively correlated to the infiltration of acquired immunocytes and negatively associated with the infiltration of innate immunocytes in lung adenocarcinoma.^18^ In this study, we identified NCAPH as an immunoregulation factor. Elevated NCAPH expression was positively related to the infiltrated CD8 T cells, Tregs and macrophages in ccRCC. NCAPH overexpression tolerated ccRCC cells to CD8 T cells‐mediated cytolysis and promoted CD8 T cell exhaustion by increasing PD1 overexpression. Moreover, we also found that NCAPH promoted PD‐L1 expression by inhibiting the degradation of β‐catenin in ccRCC cells, resulting in immune tolerance of ccRCC. Besides, NCAPH overexpression promoted resistance to anti‐PD‐1 therapy in vivo. In short, our findings revealed for the first time the function of NCAPH in immune regulation, and targeting NCAPH may be an effective treatment for ccRCC.

Metabolic reprogramming is one of the hallmarks of a wide variety of tumours, including ccRCC. Glycolysis produces large amounts of energy needed by tumour cells and increases the production of lactic acid.^44^ The release of lactic acid can form an acidic extracellular microenvironment, which further promotes aggressiveness of tumour cells.^45^ Moreover, glycolysis also plays a crucial role in tumour immune escape. The acidic extracellular microenvironment damages the recognition of immune cells to cancer cells.^46^ Production of lactic acid also blunts tumour immunosurveillance by T cells.^47^, ^48^, ^49^ In our findings, we found that NCAPH promoted aerobic glycolysis by stabilizing β‐catenin protein and upregulated NCAPH in ccRCC cells would induce CD8 T cells exhaustion by enhancing PD1 expression. Considering that PD1 could be increased by lactic acid,^49^, ^50^, ^51^ we inferred that NCAPH regulated CD8 T cells exhaustion by enhancing glycolysis and the release of lactic acid in ccRCC.

## 5 CONCLUSIONS

In summary, our study indicated that NCAPH could serve as an important prognostic predictor and an immunomodulator for ccRCC patients. NCAPH promoted the growth and anti‐PD‐1 resistance of ccRCC (Figure 7L). Targeting NCAPH could regard as an effective therapeutic strategy for ccRCC.

## AUTHOR CONTRIBUTIONS

Xing Zeng, Le Li and Ke Chen have given substantial contributions to the conception and the design of the manuscript; Jihua Tian, Xing Zeng, Chunhao Guo and Zhi Chen to acquisition, analysis and interpretation of the data. All authors have participated in drafting the manuscript; Dan Peng, Zhiquan Hu and Weiqiang Ruan revised it critically. All authors read and approved the final version of the manuscript.

## FUNDING INFORMATION

This work was supported by the Natural Science Foundation of Hubei Province (No. 2021CFB599).

## CONFLICT OF INTEREST

The authors declare that they have no conflict of interest.

## Supporting information


**Figure S1.** NCAPH is upregulated in ccRCC tissues and cell lines. (A) NCAPH expression in a variety of tumours was shown. (B) Kaplan–Meier plot showing the relationship between NCAPH levels and patient overall survival in TCGA KIRC. (C) Kaplan–Meier plot showing the relationship between NCAPH levels and patient disease‐free survival in TCGA KIRC. (D) NCAPH protein expression in CPTAC ccRCC samples and normal samples. ***p < 0.001. (E) NCAPH mRNA expression in HK‐2 cells and ccRCC cell lines. ***p < 0.001 versus HK‐2. (F) NCAPH protein expression in HK‐2 cells and ccRCC cell lines. ***p < 0.001 versus HK‐2. (G, H) NCAPH mRNA level in 786‐O and Caki‐1 cells with NCAPH overexpression or depletion. ***p < 0.001 versus vector; ^###^ *p* < 0.001 versus sh‐NC.
**Figure S2.** FOXP3 is increased and related to OS in KIRC. (A) The expression of PAX5, YY1, STAT4 and FOXP3 in TCGA KIRC samples. ***p < 0.001. (B) The correlation between STAT4 levels and NCAPH levels in KIRC. (C) The correlation between FOXP3 levels and NCAPH levels in KIRC. (D) Kaplan–Meier plot showing the relationship between FOXP3 levels and patient overall survival in TCGA KIRC. (E) Kaplan–Meier plot showing the relationship between STAT4 levels and patient overall survival in TCGA KIRC.
**Figure S3.** METTL3‐IGF2BP3 axis affects the stability of NCAPH mRNA. (A) The m6A modification of NCAPH mRNA in 786‐O and Caki‐1 cells after METTL3 depletion. ***p < 0.001 versus sh‐NC. (B) The stability of NCAPH mRNA in ccRCC cells with METTL3 depletion. ***p < 0.001 versus sh‐NC. (C) NCAPH expression in ccRCC cells with METTL3 depletion. ***p < 0.001 versus sh‐NC. (D) The stability of NCAPH mRNA in ccRCC cells with IGF2BP3 depletion. ***p < 0.001 versus sh‐NC. (E) NCAPH expression in ccRCC cells with IGF2BP3 depletion. ***p < 0.001 versus sh‐NC. (F) NCAPH expression in ccRCC cells with YTHDC1 depletion. (G) The stability of NCAPH mRNA in ccRCC cells with YTHDC1 depletion. (H) The distribution of NCAPH mRNA in nucleus and cytoplasm in ccRCC cells with YTHDC1 depletion. ***p < 0.001 versus sh‐NC.
**Figure S4.** NCAPH is related to immunoregulation. (A–F) The correlation between NCAPH expression and TILs, immunoinhibitors, immunostimulators, MHCs, chemokines and receptors in a variety of tumours from TISIDB. (G) The correlation between NCAPH expression and infiltrated CD8 T cells, CD4 T cells, B cells, Tregs and macrophages in KIRC from TIMER.
**Table S1.** The correlation between NCAPH expression and clinicopathological characteristics in 87 cRCC patients.
**Table S2.** The sequences of primers and oligonucleotides used in this study.

## Data Availability

The datasets used and analysed during the current study are available from the corresponding author upon reasonable request.
