# Supplementary material for: Non‐SMC condensin I complex subunit H participates in anti‐programmed cell death‐1 resistance of clear cell renal cell carcinomas
Source: Cell Prolif. 2023 Jan 15;56(7):e13400. doi: 10.1111/cpr.13400 (PMC10334267; doi:10.1111/cpr.13400)
Supplement: Supplementary file 1 — Figure S1. NCAPH is upregulated in ccRCC tissues and cell lines. (A) NCAPH expression in a variety of tumours was shown. (B) Kaplan–Meier plot showing the relationship between NCAPH levels and patient overall survival in TCGA KIRC. (C) Kaplan–Meier plot showing the relationship between NCAPH levels and patient disease‐free survival in TCGA KIRC. (D) NCAPH protein expression in CPTAC ccRCC samples and normal samples. ***p < 0.001. (E) NCAPH mRNA expression in HK‐2 cells and ccRCC cell lines. ***p < 0.001 versus HK‐2. (F) NCAPH protein expression in HK‐2 cells and ccRCC cell lines. ***p < 0.001 versus HK‐2. (G, H) NCAPH mRNA level in 786‐O and Caki‐1 cells with NCAPH overexpression or depletion. ***p < 0.001 versus vector; ### p < 0.001 versus sh‐NC. Figure S2. FOXP3 is increased and related to OS in KIRC. (A) The expression of PAX5, YY1, STAT4 and FOXP3 in TCGA KIRC samples. ***p < 0.001. (B) The correlation between STAT4 levels and NCAPH levels in KIRC. (C) The correlation between FOXP3 levels and NCAPH levels in KIRC. (D) Kaplan–Meier plot showing the relationship between FOXP3 levels and patient overall survival in TCGA KIRC. (E) Kaplan–Meier plot showing the relationship between STAT4 levels and patient overall survival in TCGA KIRC. Figure S3. METTL3‐IGF2BP3 axis affects the stability of NCAPH mRNA. (A) The m6A modification of NCAPH mRNA in 786‐O and Caki‐1 cells after METTL3 depletion. ***p < 0.001 versus sh‐NC. (B) The stability of NCAPH mRNA in ccRCC cells with METTL3 depletion. ***p < 0.001 versus sh‐NC. (C) NCAPH expression in ccRCC cells with METTL3 depletion. ***p < 0.001 versus sh‐NC. (D) The stability of NCAPH mRNA in ccRCC cells with IGF2BP3 depletion. ***p < 0.001 versus sh‐NC. (E) NCAPH expression in ccRCC cells with IGF2BP3 depletion. ***p < 0.001 versus sh‐NC. (F) NCAPH expression in ccRCC cells with YTHDC1 depletion. (G) The stability of NCAPH mRNA in ccRCC cells with YTHDC1 depletion. (H) The distribution of NCAPH mRNA in nucleus and cytoplasm in [file CPR-56-e13400-s001.docx]

**Figure S1**

**
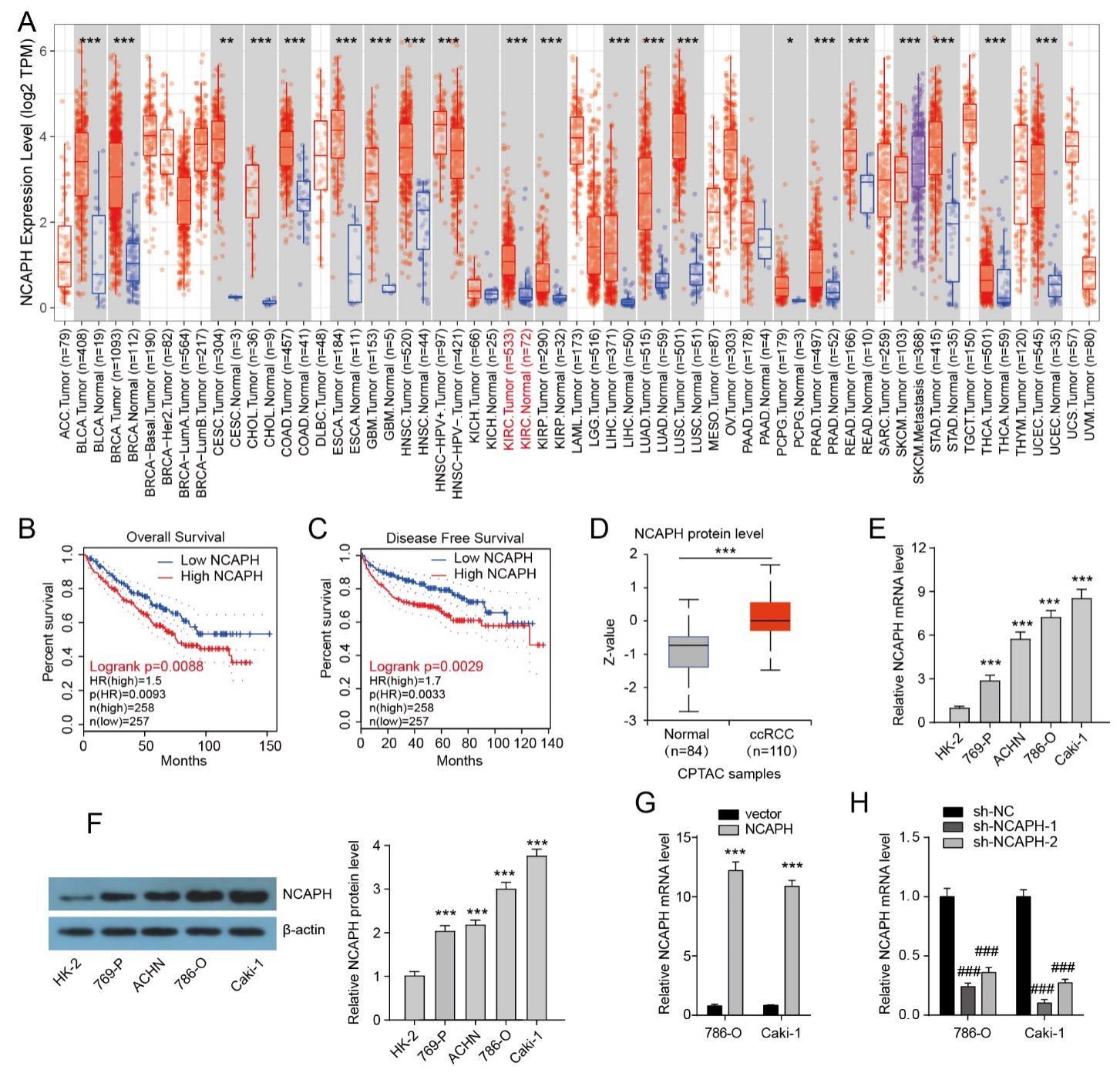
**

**Figure S1 NCAPH is upregulated in ccRCC tissues and cell lines.** (A) NCAPH expression in a variety of tumors was shown. (B) Kaplan-Meier plot showing the relationship between NCAPH levels and patient overall survival in TCGA KIRC. (C) Kaplan-Meier plot showing the relationship between NCAPH levels and patient disease-free survival in TCGA KIRC. (D) NCAPH protein expression in CPTAC ccRCC samples and normal samples. ***p<0.001. (E) NCAPH mRNA expression in HK-2 cells and ccRCC cell lines. ***p<0.001 vs HK-2. (F) NCAPH protein expression in HK-2 cells and ccRCC cell lines. ***p<0.001 vs HK-2. (G-H) NCAPH mRNA level in 786-O and Caki-1 cells with NCAPH overexpression or depletion. ***p<0.001 vs vector; ###p<0.001 vs sh-NC.

**Figure S2**

**
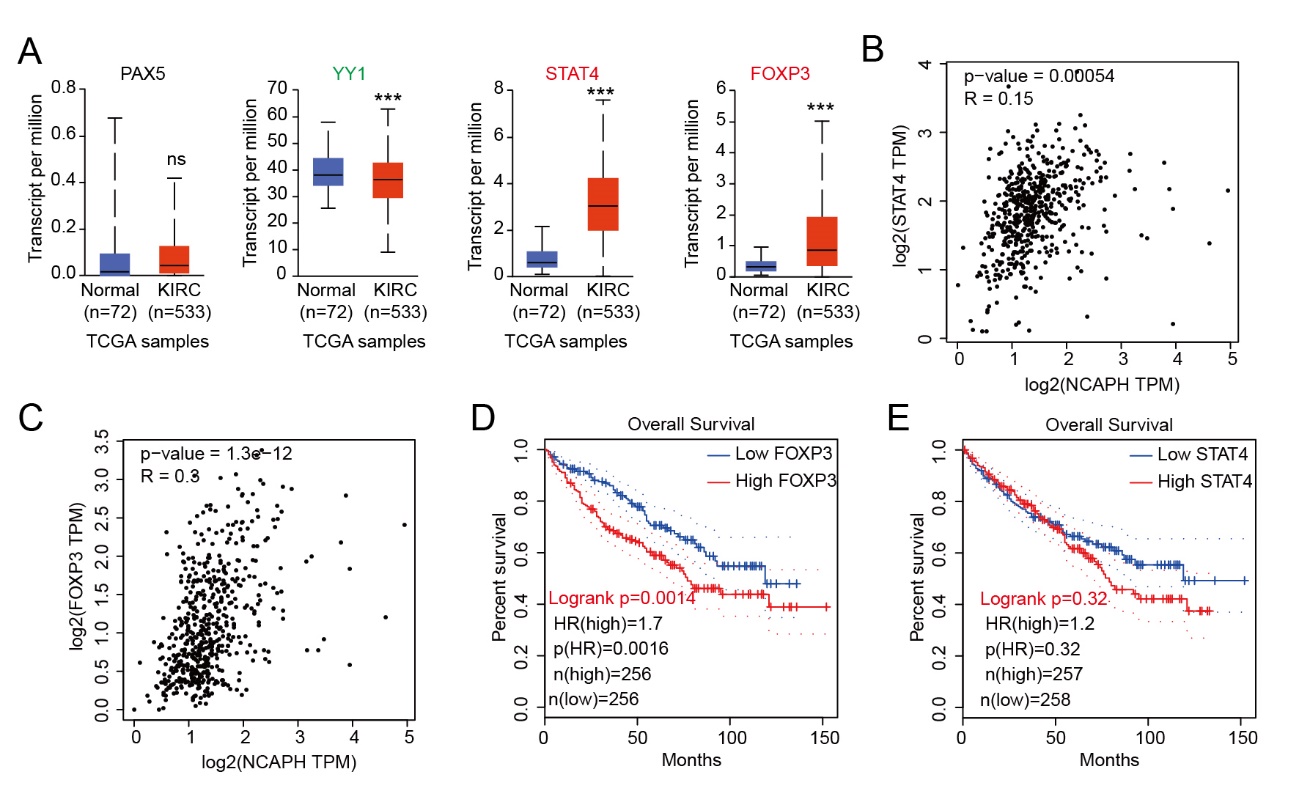
**

**Figure S2 FOXP3 is increased and related to OS in KIRC.** (A) The expression of PAX5, YY1, STAT4, and FOXP3 in TCGA KIRC samples. ***p<0.001. (B) The correlation between STAT4 levels and NCAPH levels in KIRC. (C) The correlation between FOXP3 levels and NCAPH levels in KIRC. (D) Kaplan-Meier plot showing the relationship between FOXP3 levels and patient overall survival in TCGA KIRC. (E) Kaplan-Meier plot showing the relationship between STAT4 levels and patient overall survival in TCGA KIRC.

**Figure S3**


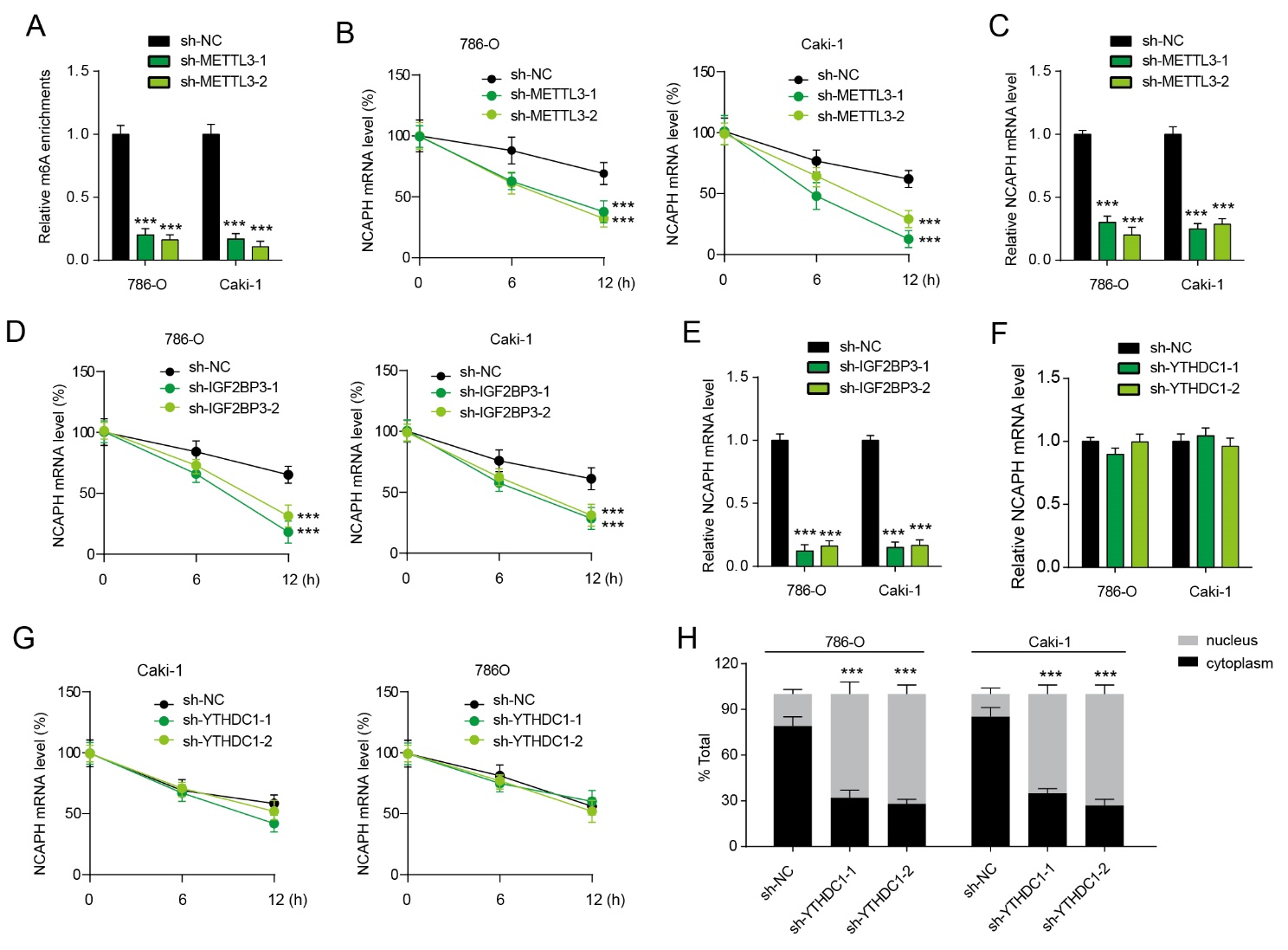


**Figure S3 METTL3-IGF2BP3 axis affects the stability of NCAPH mRNA.** (A) The m6A modification of NCAPH mRNA in 786-O and Caki-1 cells after METTL3 depletion. ***p<0.001 vs sh-NC. (B) The stability of NCAPH mRNA in ccRCC cells with METTL3 depletion. ***p<0.001 vs sh-NC. (C) NCAPH expression in ccRCC cells with METTL3 depletion. ***p<0.001 vs sh-NC. (D) The stability of NCAPH mRNA in ccRCC cells with IGF2BP3 depletion. ***p<0.001 vs sh-NC. (E) NCAPH expression in ccRCC cells with IGF2BP3 depletion. ***p<0.001 vs sh-NC. (F) NCAPH expression in ccRCC cells with YTHDC1 depletion. (G) The stability of NCAPH mRNA in ccRCC cells with YTHDC1 depletion. (H) The distribution of NCAPH mRNA in nucleus and cytoplasm in ccRCC cells with YTHDC1 depletion. ***p<0.001 vs sh-NC.

**Figure S4**

**
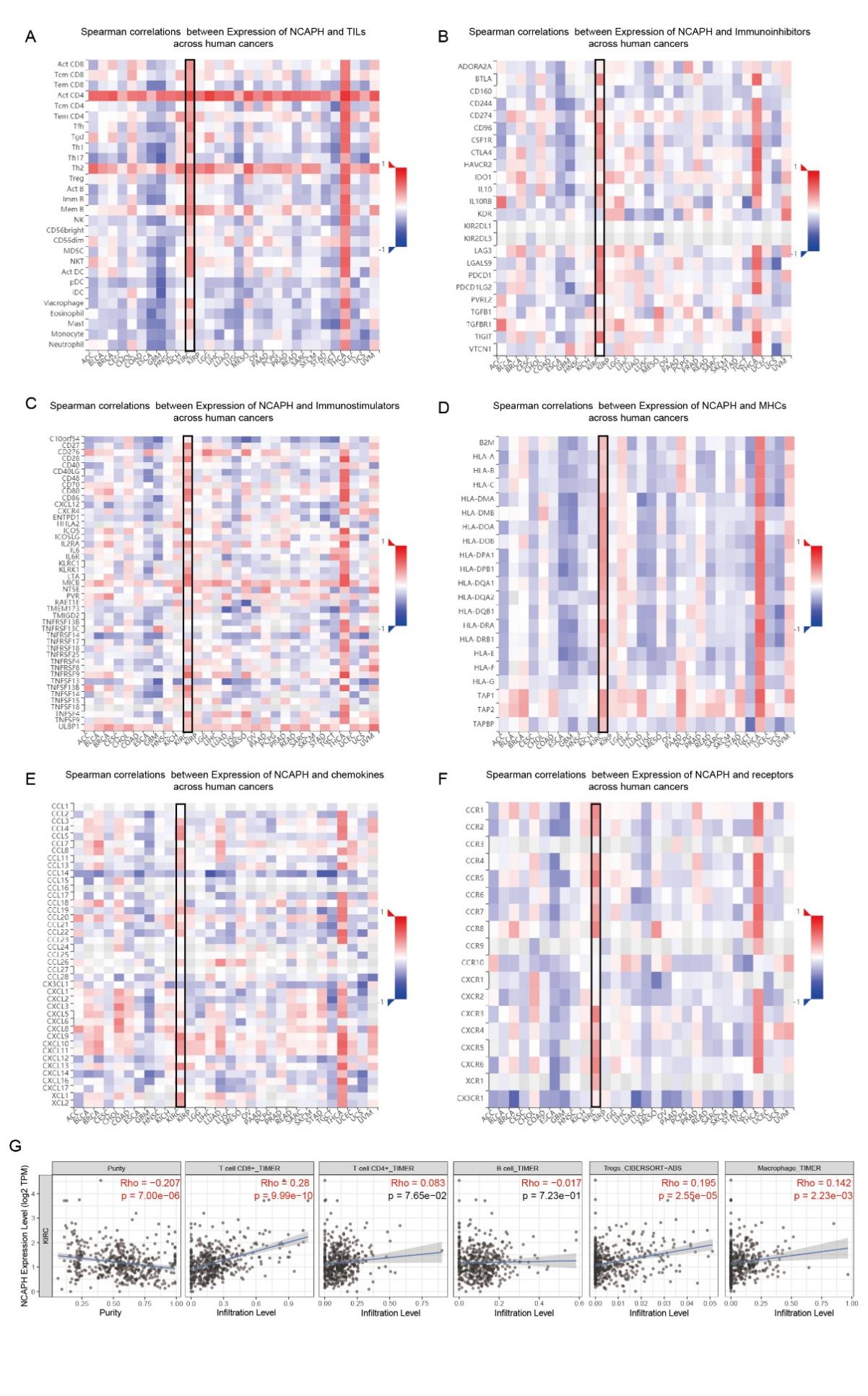
**

**Figure S4 NCAPH is related to immunoregulation.** (A-F) The correlation between NCAPH expression and TILs, immunoinhibitors, immunostimulators, MHCs, chemokines, and receptors in a variety of tumors from TISIDB. (G) The correlation between NCAPH expression and infiltrated CD8 T cells, CD4 T cells, B cells, Tregs, and macrophages in KIRC from TIMER.

**Supplementary Table 1.**

The correlation between NCAPH expression and clinicopathological characteristics in 87 cRCC patients.

| **Characteristics** | **Total** | **NCAPH High group** | **NCAPH Low group** | ***p*-value** |
| --- | --- | --- | --- | --- |
| **Age(years)** |  |  |  | 0.655 |
| ≥60 | 53 | 33 | 20 |  |
| <60 | 34 | 19 | 15 |  |
| **Gender** |  |  |  | 0.823 |
| Male | 56 | 34 | 22 |  |
| Female | 31 | 18 | 13 |  |
| **Size(cm)** |  |  |  | **0.030** |
| <4.5 | 47 | 23 | 24 |  |
| ≥4.5 | 40 | 29 | 11 |  |
| **Histologic grades** |  |  |  | **0.039** |
| 1+2 | 66 | 35 | 31 |  |
| 3+4 | 21 | 17 | 4 |  |
| **TNM Stage** |  |  |  | **0.012** |
| I | 70 | 37 | 33 |  |
| II/III | 17 | 15 | 2 |  |

**Supplementary Table 2.**

The sequences of primers and oligonucleotides used in this study.

| **qRT-PCR Primer sequences** | | |
| --- | --- | --- |
| β-actin | Forward | 5'-TCCATCATGAAGTGTGACGT-3' |
|  | Reverse | 5'-GAGCAATGATCTTGATCTTCAT-3' |
| NCAPH | Forward | 5'-CTGATGGAAGTGCTACTGAAATGG-3' |
|  | Reverse | 5'-TCTGAAACATGGGATCAATCTCAC-3' |
| FOXP3 | Forward | 5'-CCTACCCACTGCTGGCAAAT-3' |
|  | Reverse | 5'-CCTGGCAGTGCTTGAGGAA-3' |
| METTL3 | Forward | 5'-GAAAGACTATCTCCTGGCACTC-3’ |
|  | Reverse | 5'-GTACCTTTGCTTGAACCGTG-3’ |
| IGF2BP3 | Forward | 5'‐GCACTTCCCTTTGTTGTAGTC-3' |
|  | Reverse | 5'‐AGCACTTCCCTTAGGTTACTC-3' |
| YTHDC1 | Forward | 5'‐ACGATACCAGGAAGTGGAC-3' |
|  | Reverse | 5'‐CTCACATAATCATTGTAGGACCC-3' |
| PD-L1 | Forward | 5′-GCCGAAGTCATCTGGACAAG-3’ |
|  | Reverse | 5′-TCTCAGTGTGCTGGTCACAT-3’ |
| GLUT1 | Forward | 5'‐CGGGCCAAGAGTGTGTGCTAAA-3' |
|  | Reverse | 5'‐TGACGATACCGGAGCCAATG-3' |
| LDHA | Forward | 5'‐GGCCTGTGCCATCAGTATCT-3' |
|  | Reverse | 5'‐GGAGATCCATCATCTCTCCC-3' |
| HK2 | Forward | 5'‐CCAGTTCATTCACATCATCAG-3' |
|  | Reverse | 5'‐CTTACACGAGGTCACATAGC-3' |
| PKM2 | Forward | 5'‐GACTGCCTTCATTCAGACCCA-3' |
|  | Reverse | 5'‐GGGTGGTGAATCAATGTCCAG-3' |
| PD1 | Forward | 5′-ATGCAGATCCCACAGGCGCC-3′ |
|  | Reverse | 5′-TCAGAGGGGCCAAGAGCAGTG-3′ |
| **ChIP-qPCR Primer sequences** | | |
| NCAPH promoter | Forward | 5'-CCTTACGCTGAAGATTTGAAGG-3' |
|  | Reverse | 5'-GGATTATAGGCATGAGCCAC-3' |
| **shRNA sequence** | | |
| Sh-NC | 5'‐GCACTACCAGAGCTAACTCAG-3' | |
| Sh-NCAPH-1 | 5'‐TCAGAGATTCTTAAACAGAAA-3' | |
| Sh-NCAPH-2 | 5'‐TCTCCTAAATTGATCTGTTAT-3' | |
| Sh-FOXP3-1 | 5'‐CATGGACTACTTCAAGTTC-3' | |
| Sh-FOXP3-2 | 5'‐GAGAGATGGTACAGTCTCT-3' | |
| Sh-METTL3-1 | 5'‐CAAGTATGTTCACTATGAA-3' | |
| Sh-METTL3-2 | 5'‐GACTGCTCTTTCCTTAATA-3' | |
| Sh-IGF2BP3-1 | 5'‐GCTGCACTTCAGACGAATTAT-3' | |
| Sh-IGF2BP3-2 | 5'‐GCAAAGGATTCGGAAACTT-3' | |
| Sh-YTHDC1-1 | 5'‐TGGATTTGCAGGCGTGAATTA-3' | |
| Sh-YTHDC1-2 | 5'‐GGAGGAAGAAGAAGAAT-3' | |
